# Cyclic Di-Adenosine Monophosphate: A Promising Adjuvant Candidate for the Development of Neonatal Vaccines

**DOI:** 10.3390/pharmaceutics13020188

**Published:** 2021-02-01

**Authors:** Darío Lirussi, Sebastian Felix Weissmann, Thomas Ebensen, Ursula Nitsche-Gloy, Heiko B. G. Franz, Carlos A. Guzmán

**Affiliations:** 1 Department of Vaccinology and Applied Microbiology, Helmholtz Centre for Infection Research, Inhoffenstrasse 7, 38124 Braunschweig, Germany; Sebastian.Weissmann@gmx.de (S.F.W.); Carlos.Guzman@helmholtz-hzi.de (C.A.G.); 2 Women’s Clinic, Hospital Marienstift GmbH, Helmstedter Strasse 35, 38102 Braunschweig, Germany; ursula.nitsche-gloy@marienstift-braunschweig.de; 3 Department of Obstetrics and Gynecology, Women’s Clinic, Braunschweig Central Hospital, Celler Strasse 38, 38114 Braunschweig, Germany; frk_sekr@klinikum-braunschweig.de

**Keywords:** cyclic di-nucleotides (CDN), cyclic di-adenosine monophosphate (CDA), stimulator of interferon genes (STING), first dose efficacy, neonatal vaccines

## Abstract

Underdeveloped immunity during the neonatal age makes this period one of the most dangerous during the human lifespan, with infection-related mortality being one of the highest of all age groups. It is also discussed that vaccination during this time window may result in tolerance rather than in productive immunity, thus raising concerns about the overall vaccine-mediated protective efficacy. Cyclic di-nucleotides (CDN) are bacterial second messengers that are rapidly sensed by the immune system as a danger signal, allowing the utilization of these molecules as potent activators of the immune response. We have previously shown that cyclic di-adenosine monophosphate (CDA) is a potent and versatile adjuvant capable of promoting humoral and cellular immunity. We characterize here the cytokine profiles elicited by CDA in neonatal cord blood in comparison with other promising neonatal adjuvants, such as the imidazoquinoline resiquimod (R848), which is a synthetic dual TLR7 and TLR8 agonist. We observed superior activity of CDA in eliciting T helper 1 (Th1) and T follicular helper (TfH) cytokines in cells from human cord blood when compared to R848. Additional in vivo studies in mice showed that neonatal priming in a three-dose vaccination schedule is beneficial when CDA is used as a vaccine adjuvant. Humoral antibody titers were significantly higher in mice that received a neonatal prime as compared to those that did not. This effect was absent when using other adjuvants that were reported as suitable for neonatal vaccination. The biological significance of this immune response was assessed by a challenge with a genetically modified influenza H1N1 PR8 virus. The obtained results confirmed that CDA performed better than any other adjuvant tested. Altogether, our results suggest that CDA is a potent adjuvant in vitro on human cord blood, and in vivo in newborn mice, and thus a suitable candidate for the development of neonatal vaccines.

## 1. Introduction

The limited number of adjuvants licensed for human vaccines, make the development of new protective vaccines challenging and urge the search for new adjuvant molecules. Among the desired features of new adjuvants, researchers are looking for molecules that can stimulate both arms of the adaptive immune response—the humoral (antibodies) and the cellular response (cytotoxic T lymphocytes, CTL). Promising experimental adjuvants activating both arms of the immune response are members of pathogen-associated molecular patterns (PAMPs) [1]. Among them, Toll-like receptors (TLRs) agonists and C-type lectin receptors (CLRs) agonists are important adjuvant candidates. Some of them such as Lipopolysaccharide (LPS, a TLR4 agonist) and phosphatidylinositol mannosides (PIMs, -CLRs agonists) activate antigen-presenting cells (APCs) by the binding to DC-SIGN protein, which accounts for the specificity of activation on immunocompetent APCs, such as dendritic cells [2]. Other promising adjuvant candidates among PAMPs are cyclic di-nucleotides (CDNs). CDNs are acting as second messengers in microbes, therefore sensed by higher eukaryotes as an infection danger signal [3]. CDN binds to the stimulator of interferon genes (STING and other cytosolic ligands), which elicits a profuse production of type I interferon (IFN-α/β) [4,5]. Noteworthy, this type I IFN driven immunity ensues a humoral antibody response and a strong cellular CTL response [6]. These capabilities make CDNs promising adjuvants for immunization of risk populations, such as neonates, for whom antibody responses are deficient or not always correlated with vaccine protection [7].

The immature immune responses in newborns increase their susceptibility to classic and opportunistic infections, which in turn account for at least 5.5 million neonatal infections every year [8,9]. Newborns remain indeed particularly vulnerable in the first few months of their life to life-threatening infections [10]. In this regard, vaccination of newborns represents a key global strategy to overcome morbidity and mortality due to infection in early life [11]. The neonatal period of life is a window of opportunity for immunizations, especially because barriers such as skin and mucosae are more permeable to vaccine antigens, and the associated microbiota are underdeveloped [12]. Interestingly, it was demonstrated previously that neonatal vaccines could also protect against vaccine unrelated diseases, especially when they are accompanied by breastfeeding [13]. Nevertheless, due to the high frequency of immature antigen-presenting cells (APCs) [14,15] and the increased Th2 and Th17 cytokine profiles present in newborns [16], it was assumed that vaccinations during the neonatal and infant life could result in decreased immunity rather than the protection of the vaccinated newborn.

Accordingly, it was shown that the administration of a prime dose of vaccine during the neonatal or infant life period can decrease the immune response to an immunization boost later in life and therefore reduce protection [17,18]. This perception was also supported by failed vaccination trials, where the use of adjuvants capable of generating a good antibody immune response but not a cellular cytotoxic response resulted in the complete failure of the infant respiratory syncytial virus (RSV) vaccine [7]. Moreover, the use of alum-based adjuvants has been reported to increase the risk for potential adverse reactions in newborns [19,20]. It was also shown that the generation of cytotoxic T lymphocytes (CTLs) by these adjuvants is absent or very poor. In this regard, CTL responses were in turn shown to be able to inhibit RSV vaccine-enhanced disease [21]. Noteworthy, the CTL is one of the few immune response features that are functional in neonates and can be further enhanced by vaccination [22,23,24]. Therefore, the availability of adjuvants promoting Th1 and CTL responses meanwhile boosting the antibody production would represent a valuable asset for the development of neonatal vaccines.

Another aspect of newborn and infant vaccination is that current influenza vaccines are given in two doses to infant populations of age 6 to 48 months [25], whereas the same vaccine is applied only once to adults. This lack of immunogenicity in infants is in part due to mistargeting or a lack of adjuvantation in a vaccine that was produced based on the adult immune system, where humoral responses correlate with protection [26,27]. The extrapolation from the adult to the child-infant vaccination setting explains in part previous vaccination failures. In fact, parameters and standards used for adults are not suitable or optimal for the induction and evaluation of early life immunity [7]. This is a failure seen in trials carried out in other high-risk populations, such as the elderly [28].

In conclusion, due to reactogenicity and the lack of immunogenicity issues, new adjuvants tailored for neonatal and infant vaccination are urgently needed. Here, we evaluated the stimulatory capacity of cyclic di-adenosine monophosphate (CDA) on cells derived from human cord blood, and the immunogenicity and efficacy of CDA-adjuvanted formulations in neonatal mice in comparison to R848 as the “gold standard” neonatal adjuvant. Finally, we assessed the importance of the CDA-triggered type I interferon (IFN-α/β) response on B cell activation and subsequent antigen-specific IgG production.

## 2. Materials and Methods

### 2.1. Animals

WT C57BL/6 and Ifnar1−/− [29] mice were bred at the Helmholtz Centre for Infection Research (HZI). All animals were on the C57BL/6 background and were kept under specific pathogen-free conditions. All experiments were performed in compliance with the German animal protection law (TierSchG BGBl. I S 1105; 25.05.1998) and were approved by the Lower Saxony Committee on the Ethics of Animal Experiments and the responsible state office (Lower Saxony State Office of Consumer Protection and Food Safety), under permit numbers 33.11.42502-04-105/07 (08.04.2008) and 33.4-42502-04-13/1281 (30.01.2014).

### 2.2. Human Adult and Cord Blood

Adult peripheral blood for controls was collected from healthy volunteers. Cord blood was obtained by needle puncture of the umbilical cord vein right after delivery of the umbilical cord or residual placenta blood from healthy newborn donors at the Women’s Clinic (Braunschweig Central Hospital, Braunschweig, Germany). Signed informed consent from parents was obtained, ensuring that no ulterior modification of the genetic material contained or therapeutic use was involved in our study. Citrate anti-coagulated cord blood bags (Macopharma, Tourcoing, France) were stored at room temperature before the in vitro assays.

### 2.3. In Vitro Assays Using Human Blood

Adult or cord blood was mixed 1:1 with 37 °C warm RPMI 1640 medium (Gibco/Thermo Fisher, Waltham, MA, USA) and incubated with adjuvants and controls according to the previously described method [30]. Briefly, 20 µL of the adjuvants (76 µM CDA and 100 µM R848) were added to 180 µL of diluted blood in quadruplicates (in a 96 U-shape well plate) and incubated at 37 °C, 5% CO_2_ for 6 h. Supernatant fluids were assayed by flow cytometry in multiplex arrays (Biolegend, San Diego, CA, USA) or in an ELISA reader for IL-1β (eBioscience/Thermo Fisher, Waltham, MA, USA). The adjuvants CDA and R848 were used at a final concentration of 5 µg/mL (7.6 µM) and 3.5 µg/mL (10 µM), respectively.

### 2.4. Immunizations

We assessed the capability of CDA to promote antigen-specific immune responses in neonates following mucosal or subcutaneous (s.c.) vaccination. To this end, mice were immunized by s.c. or intranasal (i.n.) route in a prime/boost regime (two boost doses) with a two-week-window interval between vaccinations. Neonates (7–9 days old) were vaccinated by i.n. inoculation (6 µl) of CDA + ovalbumin (OVA), R848 + OVA, OVA alone, or control buffer. Alternatively, in one group per treatment (CDA + OVA and R848 + OVA), the neonatal dose was skipped and a prime was given at young age (21–23 days old) in order to determine the usefulness of the neonatal dose. Separately, using a different experimental setting in order to test B cell activation, neonatal mice were immunized once and sampled 14 days after vaccination. Mice received a dose consisting of 20 µg OVA (EndoGrade, Hyglos GmbH, Bernried, Germany) and 7.5 µg of CDA or 10 µg of R848 (InvivoGen, Toulouse, France). Controls received a “classical” phosphate-buffered saline solution (PBS, 10 mM Na_2_HPO_4_, 1.8 mM KH_2_PO_4_, 137 mM NaCl, and 2.7 mM KCl at pH 7.4) (Hyglos, Bernried, Germany). For T cell restimulation or pathogen challenge, after a 2–3 week interval, vaccinated mice were sacrificed, splenocytes isolated and T cell responses were tested, or animals were challenged with H1N1 PR8 influenza A/WSN/33 (WSN)-OVA(I) strain virus (*n* = 3–5 per group, experiments always done twice).

### 2.5. Lymphocyte Isolation, In Vitro Antigen Re-Stimulation, and Intracellular Cytokine Staining

Spleens lymph nodes (LNs) were homogenized by mechanical disruption on a cell strainer. Red blood cells were lysed by ammonium–chloride–potassium (ACK) lysing buffer. Lymphocytes were washed with PBS and counted for subsequent subpopulation isolation by positive selection on large (positive) selection (LS) columns (Miltenyi, Bergisch Gladbach, Germany) or in vitro restimulation. During the in vitro restimulation lymphocytes were incubated overnight in the presence of antigen (OVA, 20 µg/mL) or media (control), for subsequent intracellular cytokine/antibody staining. During the last 6 h of antigenic stimulation, cells were treated with brefeldin (5 µg/mL) and monensin (6 μg/mL). Cells were stained with antibodies specific for surface receptors and live/dead cell marker for 30 min at 4 °C (see reagents below). Cells were then washed in PBS, fixed in 1% paraformaldehyde, and permeabilized with 0.5% saponin and 0.5% bovine serum albumin (BSA) in PBS for 1 h at 4 °C. Intracellular staining with cytokine specific antibodies (see Section 2.8, Reagents) was performed in permeabilization solution for 45 min at 4 °C.

### 2.6. Viral Challenge

Under anesthesia (isoflurane), vaccinated animals and controls received i.n. 10^5^ plaque-forming units (PFUs) of the H1N1 PR8 influenza A/WSN/33 (WSN)-OVA(I) strain, which expresses the MHC class I-restricted SIINFEKL immunodominant peptide from OVA within the hemagglutinin [31]. Body weight and health parameters (e.g., piloerection and motility) were monitored daily during the first 12–15 days post-infection.

### 2.7. Data Processing

Flow cytometry data were acquired on an LSR Fortessa with FACSDiva software (BD Biosciences, San Jose, CA, USA) and were analyzed with FlowJo software (De Novo Software, Pasadena, CA, USA). Other data were analyzed with Microsoft Excel and GraphPad Prism 5 statistical software (GraphPad Software Inc., San Diego, CA, USA).

### 2.8. Reagents

BSA was purchased from Roth (Karlsruhe, Germany), saponin from Serva (Heidelberg, Germany), and live-dead UV-blue staining and CFSE were obtained from Molecular Probes (Thermo Fisher, Waltham, MA, USA). Anti-mouse antibodies: CD3 (clone 500A2, V500 conjugated) and anti-IL-2 (clone JES6-5H4, APC-Cy7 conjugated) were obtained from BD Bioscience (San Jose, CA, USA); anti-CD4 (clone RM4-5, PE-Cy7 conjugated), anti-TNF-α (clone MPG-XT22, PerCP-eF710 conjugated), anti-IL-4 (clone 11B11, APC conjugated) were purchased from eBioscience Inc. (eBioscience/Thermo Fisher, Waltham, MA, USA); and anti-CD8 (clone 53-6.7, BV650 conjugated), anti-IFN-γ (clone XMG1.2, BV711 and BV785 conjugated) were obtained from Biolegend (San Diego, CA, USA). IL-1β ELISA kit was purchased from Affymetrix (eBioscience/Thermo Fisher, Waltham, MA, USA). Multiple human cytokine detection kit, Human Th Cytokine Panel LEGENDplex was purchased from Biolegend (San Diego, CA, USA). R848 and CDA were purchased from InvivoGen (InvivoGen, Toulouse, France).

### 2.9. Statistical Analysis

The statistical significance of the differences observed between the different experimental groups was analyzed using Student’s *t*-test (GraphPad Software Inc., San Diego, CA, USA). Differences were considered significant at *p* < 0.05.

## 3. Results

### 3.1. CDA Stimulates a Th1 Cytokine Profile on Cells Derived from Human Cord Blood

**Figure 1:**
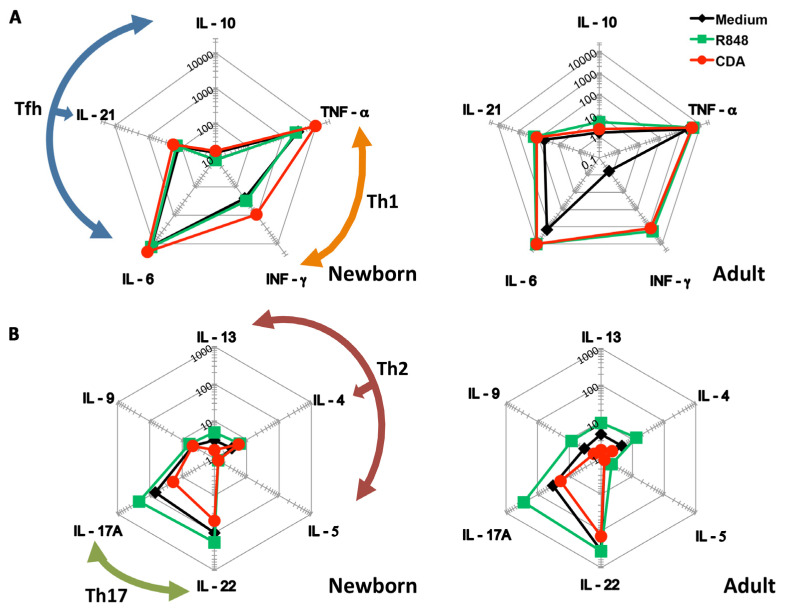
Adjuvant elicited T helper cytokine profiles on human newborn and adult blood. Human cord blood was used as a neonatal blood surrogate in vitro. Cord blood and human adult blood controls were diluted 1:1 with RPMI 1640 and incubated for 6 h in the presence of the neonatal gold standard adjuvant R848 (3.5 µg/mL/10 µM), CDA (5 µg/mL/7.6 µM), and medium control. Spider plots display cytokine values in pg/mL in supernatants of treatments for (**A**) IL-10, IL-21, and IL-6 (T follicular helper profile, Tfh), TNF-α and IFN-γ (Th1); and (**B**) IL-17 and IL-22 (T helper 17, Th17), IL-4, IL-5, and IL-13 (T helper 2, Th2) in cord blood (newborn) or adult blood. Results correspond to one out of two independent experiments.

One of the well-established methods to assess adjuvant immunogenicity in human neonates is the use of cord blood as a surrogate [30]. Therefore, to evaluate the secretory cytokine profile elicited by CDA in neonates, we incubated cord and adult blood in the presence of adjuvants and controls for 6 h. A bead-based multiplexed immunoassay array was then employed in order to detect cytokines in the supernatants. We found that TNF-α and IFN-γ (cytokines produced by T helper 1 (Th1) cells) were present at higher concentrations when the cord blood was treated with CDA as compared to R848 or medium (Figure 1A). Nevertheless, these differences were not observed when testing blood from adults. We also detected higher concentrations of IL-6, IL-21, and IL-10 (cytokines belonging to the T follicular helper (Tfh) differentiation profile). Slightly similar results were obtained when testing blood from adults (Figure 1A). Interestingly, the stimulation by CDA of cytokines produced by T helper 2 and 17 (Th2 and Th17) cells was more modest compared to what was observed after R848 treatment and in control cells from both adult and cord blood (Figure 1B). Th17 cytokines were particularly decreased after treatment with CDA, and also IL-9 was slightly decreased in cord blood samples and this difference was accentuated over the other treatments in adult blood (Figure 1B). Remarkably, CDA increased the secretion of Th1 cytokines, which are important to counteract the Th2 bias present at birth [32]. Interestingly, by using ELISA, we found that IL-1β (a pro-inflammatory cytokine) was slightly decreased in CDA-treated blood with respect to R848, in adult and in cord blood (Figure S1).

### 3.2. CDA-Adjuvanted Neonatal Dose Elicits Multiple-Cytokine Producers among Antigen-Specific T Cells

**Figure 2:**
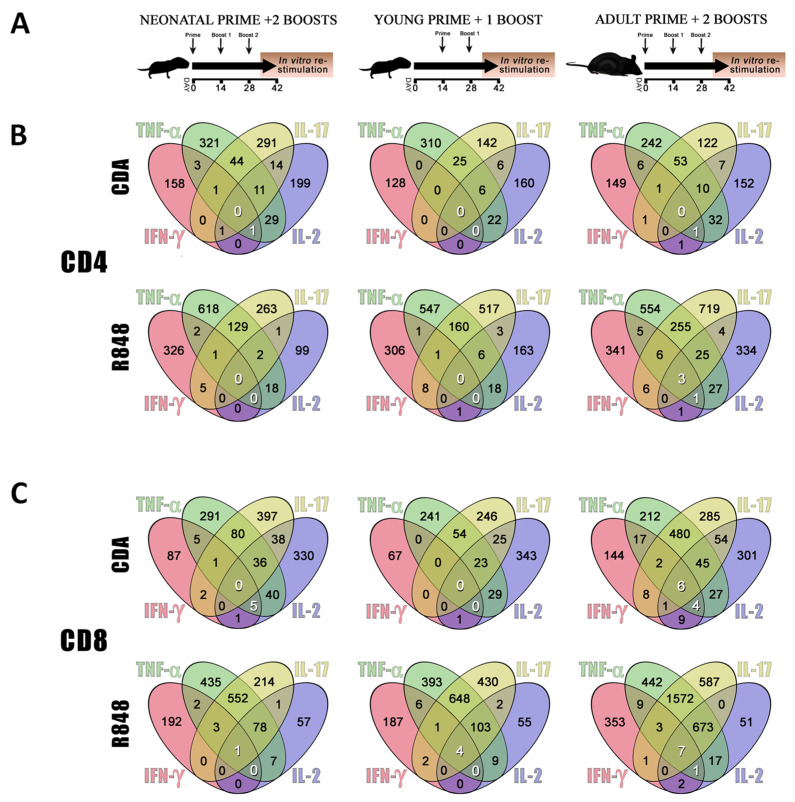
Mice antigen-specific cytokine responses to neonatal vaccination with different adjuvants. (**A**) Different vaccination strategies with ovalbumin (OVA) antigen and adjuvants (R848 and CDA). Neonatal mice received a prime (6–9 days old) followed by two boost doses (left diagram, neonatal prime + 2 boosts). Alternatively, the neonatal prime was skipped (center diagram, young prime + boost) or mice received the three doses as adult (right diagram, adult prime + 2 boosts). (**B**) Venn diagrams depicting the number of CD4^+^ T lymphocytes cytokine producers by million of cells measured by flow cytometry, after in vitro restimulation of splenocytes and surface and intracellular cytokine staining. Numbers in white are quadruple and triple cytokine producers regarded as indicators of maximal vaccine efficacy (i.e., producers of IL-2, IFN-γ, and TNF-α). Treatments depicted in A) correspond to the diagrams in the column below, where it is R848 or CDA adjuvantation. (**C**) Venn diagrams depicting the number of CD8^+^ T lymphocytes positive for individual or multiple intracellular cytokine staining. White numbers are quadruple or triple cytokine producers by million of cells analyzed. Results are average of 3–5 mice per group.

The above-mentioned in vitro approach is very useful as an initial screening for the evaluation of cytokine profiles promoted by candidate adjuvants after stimulation of different types of cells. However, it does not allow accurate prediction of important effector functions of an adjuvant, such as CTL stimulation capabilities, target cell subpopulations, and capacity to confer protective immunity. Therefore, neonatal mice were immunized to assess the in vivo performance of CDA in an active vaccination setting. To this end, a group of mice received a neonatal prime at day 7 after birth (day 0 of the vaccination schedule) followed by two boosts on days 14 and 28 of the vaccination schedule. A second group received the prime at day 21 after birth (young age, day 14 of the vaccination schedule), followed by one boost on day 28, and the third group of mice received the prime at adult age (more than 8 weeks) followed by two boosts 14 and 28 days later (Figure 2A). To assess vaccination effectivity, the frequencies of cytokine producers among the CD4^+^ and CD8^+^ T cell populations were evaluated by intracellular cytokine staining and flow cytometry. The production of multiple cytokines (especially IL-2, IFN-γ, and TNF-α) by T cells has been described to correlate with vaccine protective efficacy [33]. Thus, the frequency of T cells producing single cytokines or combinations (positive events/million) was analyzed by flow cytometry (Figure 2B,C). The same vaccination schedule was used for groups receiving R848 as the “gold standard” adjuvant that has been extensively used to promote neonatal immunity [34,35,36]. Although R848 has been claimed to elicit potent immunity when used in neonates [35,37,38,39,40], we found that in our experimental setting the use of R848 did not significantly increase the frequency of multifunctional T cells expressing IFN-γ, TNF-α, and IL-2. Moreover, the presence of CD4^+^ T cells that produce any possible combination of the four analyzed cytokines (IFN-γ, TNF-α, IL-17, and IL-2) was indeed reduced when a neonatal dose of R848 was used as compared to the treatment where this dose was skipped or in adult controls (Figure 2A). In contrast, the use of a neonatal dose of CDA-adjuvanted vaccine increased the frequency of triple, double, and single cytokine producers, when compared with the group where the neonatal dose was skipped. Interestingly, neonatal priming resulted in better stimulation of multifunctional T cells than priming at a later age when CDA was used as an adjuvant (Figure 2B, upper part). A subcutaneous (s.c.) vaccination at days 6–9 with OVA + CDA or alum also resulted in an increase of triple cytokine producers among CD4^+^ T cells from the CDA + OVA vaccinated animals over the other treatments (Figure S2).

In order to characterize more accurately the neonatal cellular immune response, the production of intracellular cytokines was measured in CD8^+^ T cells. Multifunctional CD8^+^ T cells were only stimulated when mice received neonatal priming with OVA + CDA (Figure 2C, upper left). When animals were vaccinated as adults, the responses were stronger for any particular cytokine or its combinations (Figure 2C, upper right). In contrast, when R848 was used as a neonatal golden standard adjuvant, multifunctional CD8^+^ T cells were reduced as compared to the group where the neonatal dose was skipped. In a similar fashion as for the groups receiving CDA as an adjuvant, when mice were vaccinated as adults the responses were stronger for any considered cytokine combination (Figure 2C, lower part). It is important to note that when the capacity of neonatal priming to generate multifunctional CD8^+^ T cells producing IFN-γ, TNF-α, and IL-2 was assessed (numbers highlighted in white), although both adjuvants promoted similar immune responses in adults, only CDA increased the frequency of triple cytokine producers when administered at neonatal age (Figure 2B,C). A general gating strategy for the analysis of cell subpopulations and the measurement of multiple cytokine producers after vaccination is displayed in Figure S3.

### 3.3. A CDA-Adjuvanted Neonatal Vaccine Induces B Cell Activation and Maturation

**Figure 3:**
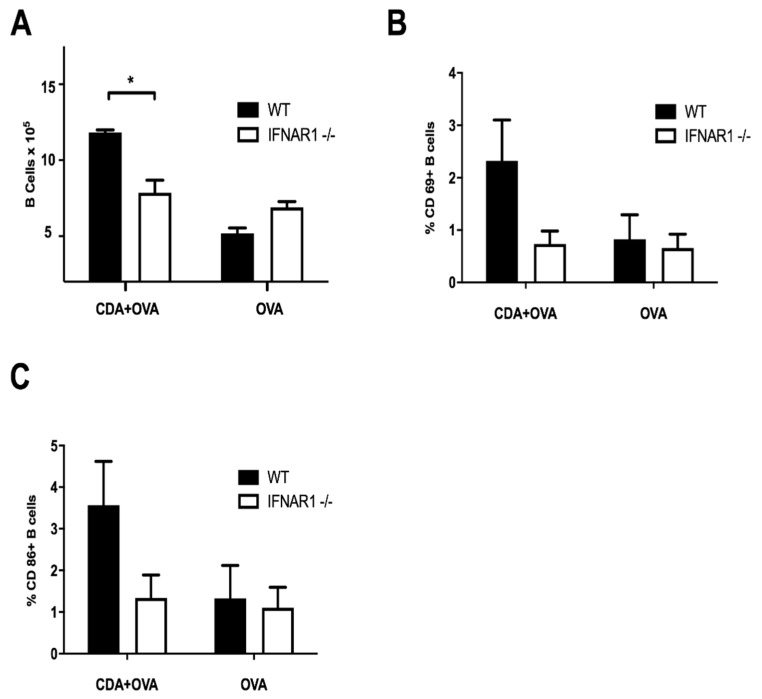
Neonatal vaccination with CDA induces B cell activation and maturation. Percentage of activated B cells after a prime-dose in neonatal mice. (**A**) Number of B cells alive after restimulation. (**B**) Frequency of CD69^+^ B cells or (**C**) CD86^+^ B cells from WT (B6) or IFNAR1−/− (IFN receptor KO) mice vaccinated with CDA + OVA or OVA alone to determine the dependence of B cell maturation on the CDA induced IFN-α/β. Results are from one representative experiment out of three independent experiments consisting of 3–5 mice per group. Differences are significant when marked (*), otherwise not significant by a non-paired Student’s *t*-test (*p* ≤ 0.05).

A key feature to determine vaccine efficacy is also the activation of B cells. Importantly, neonatal B cells are regulated by type I interferon (IFN-α/β) on their capacity to moderate inflammation upon infection via IL-10 [41]. It has also been reported that IFN-α/β is necessary for the activation and development of B cells in neonates [42]. In order to assess whether CDA-mediated generation of IFN-α/β was necessary for B cells maturation after vaccination, we vaccinated WT and IFNAR1−/− neonatal mice with CDA + OVA, OVA alone, or vehicle control. Neonatal mice were vaccinated by i.n. route at days 7–9 after birth, and euthanized 14 days after vaccination to measure the development and activation of B lymphocytes after in vitro restimulation with OVA (Section 2.5, Materials and Methods). We observed that the number of viable B cells after antigen-specific restimulation of splenocytes was significantly higher in WT mice vaccinated with CDA + OVA than in all other treatment groups (Figure 3A). Importantly, the expression of the activation marker CD69 and the surface maturation marker CD86 on the B cell subset was enhanced in WT mice vaccinated with CDA + OVA as compared with the other groups (Figure 3B,C). Although not statistically significant in neonates, the differences were maintained and acquired significance in adults after a prime and boost vaccination approach (Figure S4). This suggests that neonatal vaccination with CDA triggers IFN-α/β dependent mechanisms that are necessary for the activation and development of B cells.

### 3.4. Neonatal CDA Vaccination Promotes a Protective Immune Response in Adult Mice

**Figure 4:**
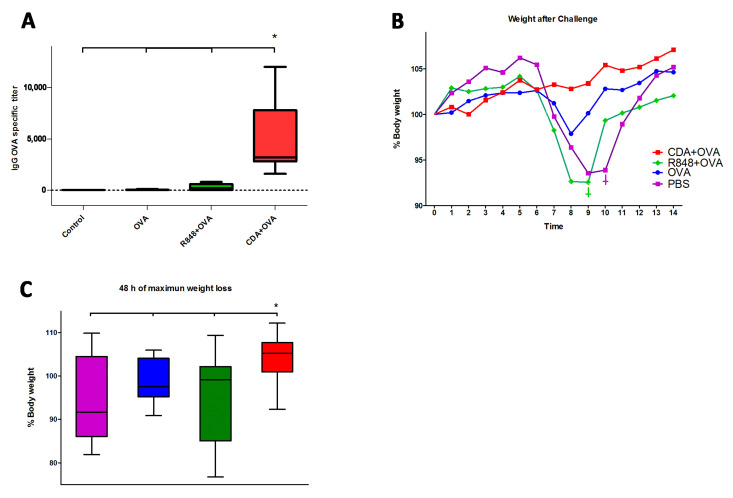
Neonatal vaccination with CDA confers protection in adult mice. Mice intranasally vaccinated with CDA + OVA, R848 + OVA, OVA, or PBS (control) were challenged by 10^5^ PFU of the H1N1 PR8 influenza A/WSN/33 (WSN)-OVA(I) virus, which expresses the SIINFEKL peptide in the hemagglutinin domain. (**A**) Antigen-specific IgG from blood serum was measured by ELISA from blood sampled previous to challenge. Mice Vaccinated with CDA + OVA as neonates displayed significantly higher titers than animals receiving the other vaccine formulations. (**B**) Weight loss was recorded 14 days after infection as an inverse correlation of protection, where dead animals were found only in the PBS and the R848 groups (inverted crosses in plot B). (**C**) The differences between groups on the 48 h of maximum weight loss were significant between CDA + OVA vaccinated animals and the other treatments. In (**A**,**C**) differences are significant (*) by a non-paired Student’s *t*-test (* *p* ≤ 0.05) performed against each other treatment/control. Results shown are average from five (PBS and OVA controls) and six (CDA and R848 treatments) mice per group, from one representative out of two experiments.

In order to assess the biological significance of our findings, neonatal mice were vaccinated using CDA as an adjuvant, and controls received R848 as a control neonatal adjuvant [43]. Mice were vaccinated according to the scheme displayed in Figure 2A, which consisted of a prime immunization given at day 7 of life (day 0 of the vaccination schedule), followed by two boosts on days 14 and 28 of the vaccination schedule. After vaccination, mice were challenged with a recombinant OVA-peptide expressing H1N1 influenza virus [31], and mice were monitored for 14 days after infection. In order to determine if the humoral response is stronger upon neonatal vaccination with CDA, OVA-specific IgG titers were measured in mice sera before the H1N1 challenge. Neonatal vaccination with CDA + OVA elicited the highest titers of antigen-specific IgG, being significantly higher not only in comparison to the OVA alone and PBS controls and in respect to the titers observed in mice vaccinated using R848 as an adjuvant (Figure 4A). Noteworthy, one mouse per group died in the control group and in the R848 group after challenge, whereas no deaths were observed in the OVA only or in the CDA + OVA group. Mice vaccinated with CDA + OVA maintained and gained weight over the 14 days after challenge (Figure 4B). In contrast, mice vaccinated with the TLR7-8 agonist R848, OVA alone, or PBS lost weight during the treatment, particularly between day 7 and 9 post-challenge (Figure 4B,C). The protection conferred by CDA was also significantly higher than the one conferred by the CDA-based vaccination where the neonatal dose was skipped (data not shown). The differences in protection between CDA and the other adjuvants and controls were significant during the 48 h of maximal weight loss (Figure 4C).

## 4. Discussion

Due to the prevalence of upper airway infections during the first year of life, and the trauma associated with needle vaccination, i.n. delivery of vaccines is an attractive immunization strategy. Therefore, we use a mucosal adjuvant administered i.n., whose vaccination potency is not restricted to this administration route since sub-cutaneous vaccination was also performed here and in other works [44,45]. We had previously shown that CDA is a potent naturally occurring adjuvant that is able to elicit strong immune activation, leading to not only humoral but also cellular responses [46]. The cellular response also encompasses the induction of a CTL component, whose presence and efficacy depend on the stimulation of IFN-α/β, and its cross-priming capabilities [44]. Thus, one of the main advantages of the CDA over most vaccine adjuvants is its capacity to promote humoral antibody responses as well as cellular CTL responses. In this regard, neonatal APCs were shown to be competent in MHC class I antigenic processing and presentation [47], and in further stimulation of CTL responses [22,23,24].

In order to determine which cytokine profile is promoted by CDA vaccination, we assessed the cytokine production pattern elicited after CDA stimulation of human adult blood and cord blood (as a neonate surrogate) in vitro. We observed that CDA induces higher concentrations of Th1 and Tfh cytokines than controls and the golden standard adjuvant R848. These findings were further confirmed in vivo by using a murine experimental model. Because first dose effectiveness is a controversial issue on infant vaccination [18], we measured the differences in cytokine secretion when the neonatal dose was skipped in vivo. In order to measure the importance of a neonatal dose, subsequent vaccination boosts must be administered. Therefore, we selected a vaccination schedule of a prime and two boosts, where in some cases just the neonatal dose was skipped. The obtained results demonstrated that while a neonatal dose containing the “gold standard” candidate neonatal adjuvant R848 has negative effects on multiple cytokine producers, a neonatal dose adjuvanted with CDA has beneficial effects as a prime. Moreover, we were able to corroborate that the cytokine profiles detected in CDA-treated human cord blood were maintained in vivo for multiple cytokine producers. This effect is an advantageous feature of CDN over TLR agonists because the former has its signaling pathways and molecular targets conserved between mice and humans, whereas TLR agonists do not [48]. We demonstrated here that these properties are conserved at early ages (neonatal period) and that CDA performs better than other proposed neonatal adjuvants.

When evaluating the humoral response, we showed that neonatal vaccination with CDA elicits higher titers of antigen-specific IgG than those observed using R848. In agreement with this enhanced antibody production, we demonstrated that CDA promotes the activation of B cells at neonatal and adult ages, early upon vaccination. The activation of B cells by CDA was also dependent on type I IFN signaling. In addition to R848, other TLR agonists such as mono-phosphoryl lipid A (MPLA) [37] and CpG [49] showed promising results as neonatal adjuvants. Neonatal vaccination with CDA + OVA promoted higher antigen-specific IgG titers and protection against challenge than what we observed in mice receiving MPLA or CpG as adjuvants (data not shown).

It has been shown that although most of the adaptive immunity is deficient in newborns [32,50], CTL responses are normally developed at the neonatal age [22,47]. Therefore, adjuvants that promote the generation of CTL responses are highly desirable for neonatal vaccination. The results presented here showed that CDA is able to promote a protective CTL response capable of protecting against a genetically modified influenza virus that expresses an MHC class I-restricted CD8 epitope (SIINFEKL), and that this CTL response is dependent on the administration of the neonatal prime dose. In our experimental system, the neonatal administration of the TLR 7/8 agonist R848 as adjuvant resulted in a detrimental effect post-immunization. In contrast, the effect of CDA-mediated immune stimulation triggers protective immunity. Altogether, our data support a promising role of CDA as a neonatal adjuvant, which can be exploited as a tool in vaccines for newborns and infants in order to reduce antigen load or the number of doses required to achieve protective immunity.

## 5. Patents

As a result of the present research, Lirussi, D., Guzmán, C.A., Ebensen T. and Weissmann, S.F. are named as inventors in a patent covering the use of CDA as a neonatal adjuvant (EP 19193982), which was previously patented covering the use of CDA as an adjuvant (PCT/EP 2006010693) naming Guzmán, C.A. and Ebensen T. as inventors. This does not alter our adherence to *Pharmaceutics* policies on sharing data.

## Supplementary Materials

The following are available online at https://www.mdpi.com/1999-4923/13/2/188/s1, Figure S1: IL-1β cytokine profile on human cord blood. IL-1β content in supernatants of treated samples was assessed by ELISA. Values correspond to untreated controls or samples treated with adjuvants (R848 or CDA), either cord blood (neonates, white bars) or adult blood (black bars), Figure S2: Frequency of CD4 T lymphocytes positive for different cytokines by flow cytometry after intracellular cytokine staining. Neonatal mice (6–9 days) were immunized by s.c. route with CDA + OVA, alum + OVA, OVA, or vehicle (negative control). After in vitro restimulation with OVA antigen, the frequency of TNF-α, IFN-γ, and IL-2 triple producers was measured. Results from a single experiment with 3 animals per group. Differences were not significant by a non-paired Student’s *t*-test (*p* ≤ 0.05), Figure S3: General gating strategy for dissection of T and B cell subsets and intracellular cytokine staining/maturation markers. A general gating strategy used to dissect diverse cell subsets among immune cells, as T CD4 and CD8, B cells, and cytokine production by intracellular staining is displayed. The first two steps ensure the gating of single cells by the main population distributed obliquely on forward scatter H (height) and A (area), as well as cell population distributed horizontally inside scatter W (wide) and A (area). Once a single population is gated, then, a live/dead marker is used to gate only on cells that are alive by the time of staining. Gating on live cells will allow separating CD3+ (T cells) from CD3− (B cells among them). From CD3− cells, the CD19+ population is gated as B cells for further analysis, and from CD3+ CD4 and CD8 are gated for further analysis. By combining intracellular cytokine staining and the use of Boolean gates in Flow Jo software, the population of multiple producers of selected cytokines are measured, Figure S4: Percentage of activated B cells after prime dose vaccination in adult mice. (A) Frequency of CD69+ B cells or (B) CD86+ B cells from WT (C57BL6) or IFNAR1−/− (IFN receptor KO) mice vaccinated with CDA + OVA or OVA alone to determine the dependence of B cell maturation on CDA induced IFN-α/β. Results from one representative out of three independent experiments are shown. Differences are significant by a non-paired Student’s *t*-test (*p* ≤ 0.05).
