# Supplementary material for: Cyclic Di-Adenosine Monophosphate: A Promising Adjuvant Candidate for the Development of Neonatal Vaccines
Source: Pharmaceutics. 2021 Feb 1;13(2):188. doi: 10.3390/pharmaceutics13020188 (PMC7912751; doi:10.3390/pharmaceutics13020188)
Supplement: Supplementary file 1 [file pharmaceutics-13-00188-s001.pdf]

pharmaceutics

Supplementary Materials: Cyclic Di-Adenosine Monophosphate: A Promising Adjuvant Candidate for the Development of Neonatal Vaccines

Darío Lirussi, Sebastian Felix Weissmann, Thomas Ebensen, Ursula Nitsche-Gloy, Heiko B. G. Franz and Carlos A. Guzmán

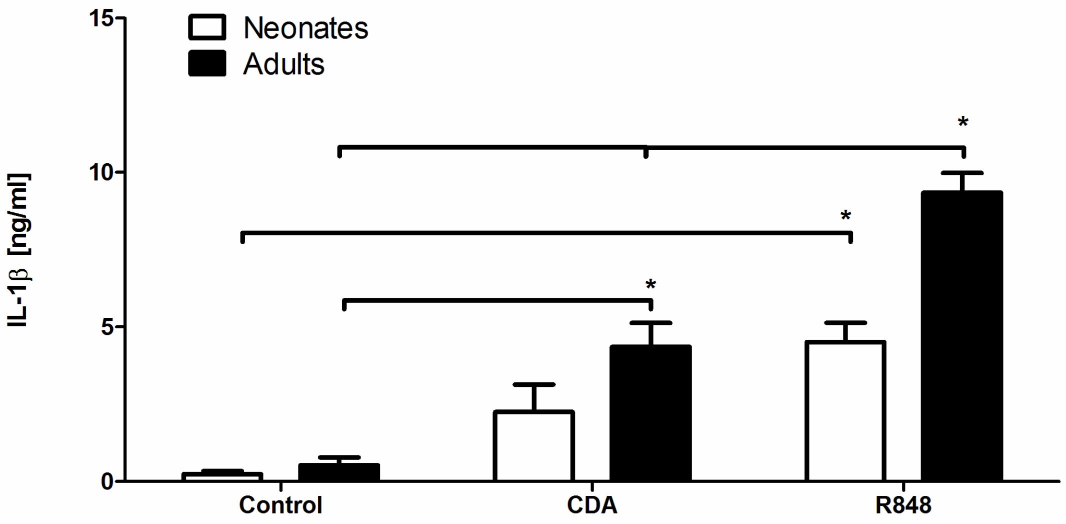

Figure S1. IL-1β cytokine profile on human cord blood. IL-1β content in supernatants of treated samples was assessed by ELISA. Values correspond to untreated controls or samples treated with adjuvants (R848 or CDA), either cord blood (neonates, white bars) or adult blood (black bars) (* $p \leq 0.05$).

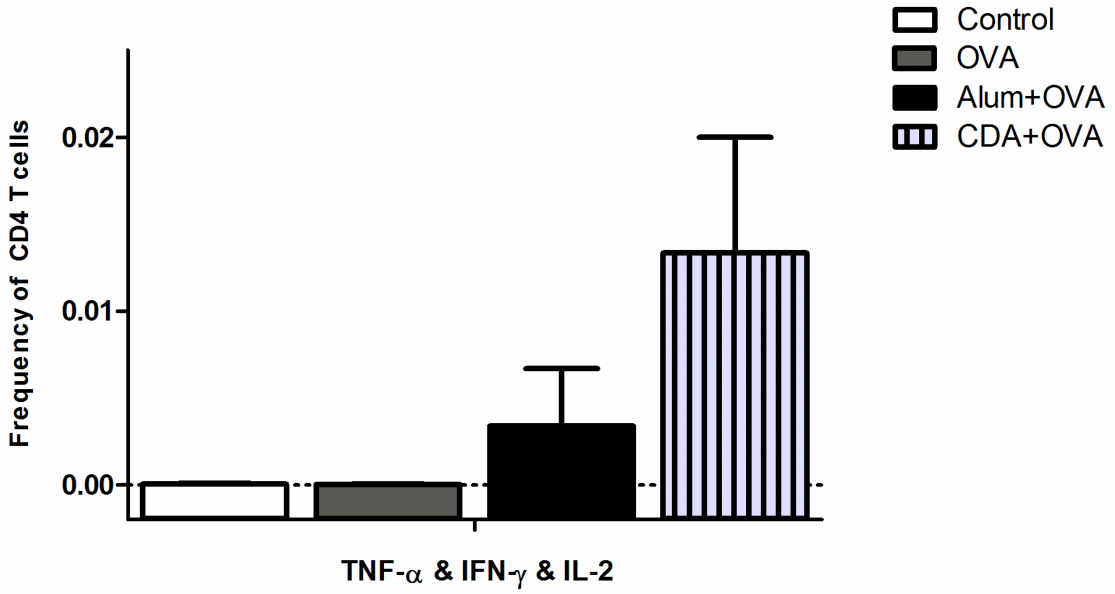

Figure S2. Frequency of CD4 T lymphocytes positive for different cytokines by flow cytometry after intracellular cytokine staining. Neonatal mice (6–9 days) were immunized by s.c. route with CDA+OVA, alum+OVA, OVA or vehicle (negative control). After in vitro re-stimulation with OVA antigen, the frequency of TNF-α, IFN-γ and IL-2 triple producers was

measured. Results from a single experiment with 3 animals per group. Differences were not significant by a non-paired student *t* test ($p \leq 0.05$).

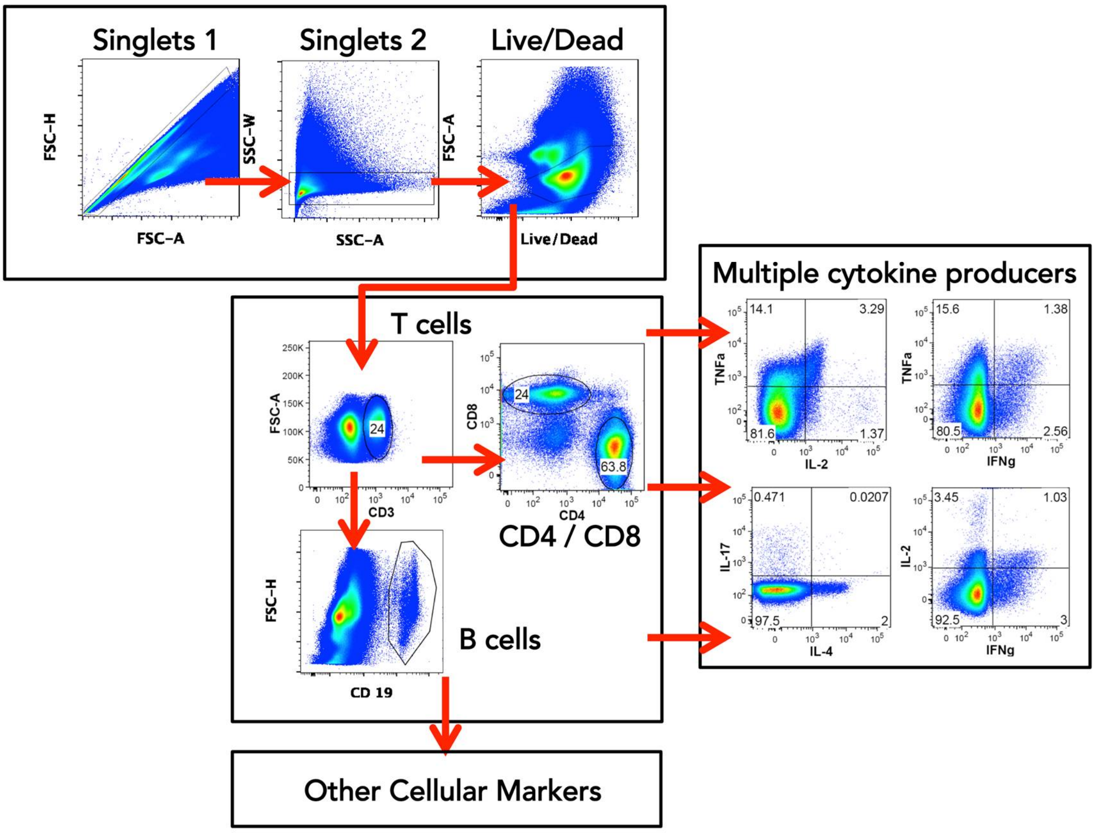

Figure S3. General gating strategy. General gating strategy for dissection of T and B cell subsets and intracellular cytokine staining/maturation markers. A general gating strategy used to dissect diverse cell subsets among immune cells, as T CD4 and CD8, B cells and cytokine production by intracellular staining is displayed. The first two steps ensure the gating of single cells by main population distributed obliquely on forward scatter H (height) and A (area), as well as cell population distributed horizontally in side scatter W (wide) and A (area). Once a singlet population is gated, then, a live/dead marker is used to gate only on cells that are alive by the time of staining. Gating on live cells will allow separating CD3+ (T cells) from CD3- (B cells among them). From CD3– cells, the CD19 + population is gated as B cells for further analysis and from CD3+ CD4 and CD8 are gated for further analysis. By combining intracellular cytokine staining and the use of Boolean gates in Flow Jo software, population of multiple producers of selected cytokines are measured.

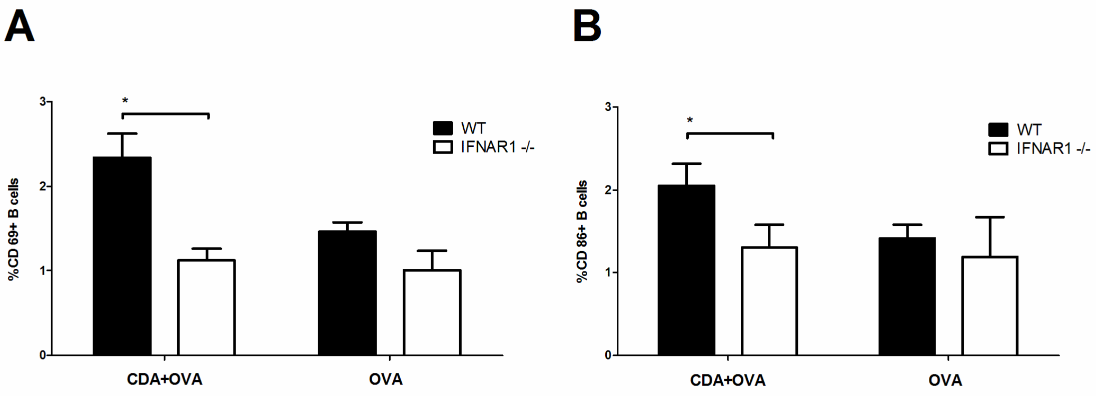

Figure S4. Percentage of activated B cells after prime dose vaccination in adult mice. **A**) Frequency of CD69+ B cells or **B**) CD86+ B cells from WT (C57BL6) or IFNAR1-/- (IFN receptor KO) mice vaccinated with CDA+OVA or OVA alone to determine the dependence of B cell maturation on CDA induced IFN-α/β. Results from one representative out of 3 independent experiments is shown. Differences are significant by a non-paired student *t* test (* $p \leq 0.05$).
